# Supplementary material for: Potential Utility of Pre-Emptive Germline Pharmacogenetics in Breast Cancer
Source: Cancers (Basel). 2021 Mar 11;13(6):1219. doi: 10.3390/cancers13061219 (PMC7998388; doi:10.3390/cancers13061219)
Supplement: Supplementary file 1 [file cancers-13-01219-s001.zip › cancers-1095450-supplementary-xml/cancers-1095450-Figure S1.pdf]

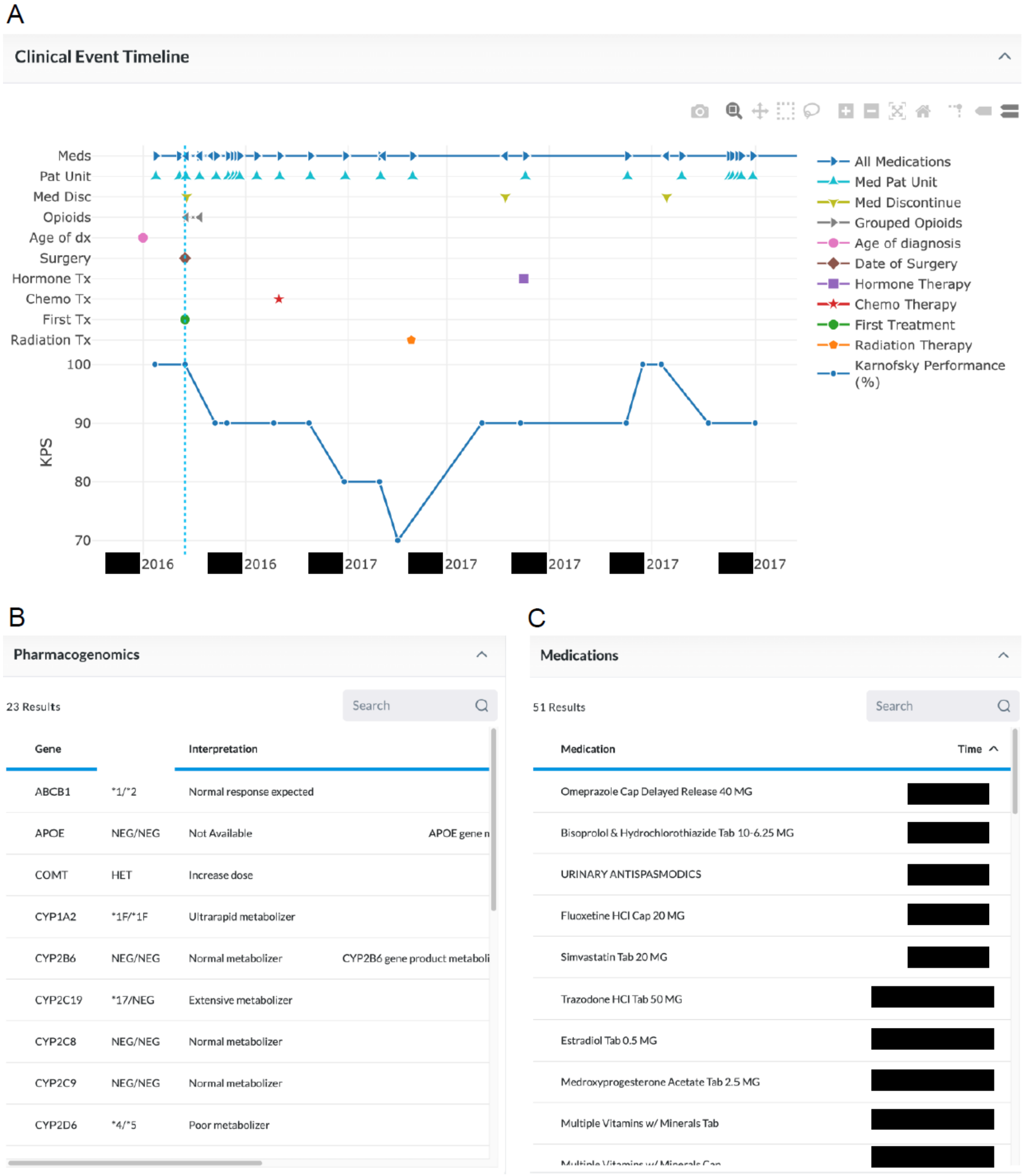

Gene		Interpretation	
ABCB1	*1/*2	Normal response expected	
APOE	NEG/NEG	Not Available	APOE gene n
COMT	HET	Increase dose	
CYP1A2	*1F/*1F	Ultrapid metabolizer	
CYP2B6	NEG/NEG	Normal metabolizer	CYP2B6 gene product metaboli
CYP2C19	*17/NEG	Extensive metabolizer	
CYP2C8	NEG/NEG	Normal metabolizer	
CYP2C9	NEG/NEG	Normal metabolizer	
CYP2D6	*4/*5	Poor metabolizer	

Medication	Time
Omeprazole Cap Delayed Release 40 MG	[redacted]
Bisoprolol & Hydrochlorothiazide Tab 10-6.25 MG	[redacted]
URINARY ANTISPASMODICS	[redacted]
Fluoxetine HCl Cap 20 MG	[redacted]
Simvastatin Tab 20 MG	[redacted]
Trazodone HCl Tab 50 MG	[redacted]
Estradiol Tab 0.5 MG	[redacted]
Medroxyprogesterone Acetate Tab 2.5 MG	[redacted]
Multiple Vitamins w/ Minerals Tab	[redacted]
Multiple Vitamins w/ Minerals Cap	[redacted]

Figure S1. PHC screenshot of clinical timeline view and data tables for representative breast cancer patient referenced in Case Study 1.
